# Changes in adult sex ratio in wild bee communities are linked to urbanization

**DOI:** 10.1038/s41598-019-39601-8

**Published:** 2019-03-06

**Authors:** Gordon Fitch, Paul Glaum, Maria-Carolina Simao, Chatura Vaidya, Jill Matthijs, Benjamin Iuliano, Ivette Perfecto

**Affiliations:** 1 Department of Ecology and Evolutionary Biology University of Michigan 1105 North University, Ann Arbor, MI 48109 USA; 2 New Jersey Agricultural Experiment Station Rutgers University 88 Lipman Dr, New Brunswick, NJ 08901 USA; 3 School of Environment and Sustainability University of Michigan 440 Church St, Ann Arbor, MI 48109 USA; 4 University of Wisconsin-Madison, Wisconsin Energy Institute 1552 University Ave, Madison, WI 53726 USA

## Abstract

Wild bees are indispensable pollinators, supporting global agricultural yield and angiosperm biodiversity. They are experiencing widespread declines, resulting from multiple interacting factors. The effects of urbanization, a major driver of ecological change, on bee populations are not well understood. Studies examining the aggregate response of wild bee abundance and diversity to urbanization tend to document minor changes. However, the use of aggregate metrics may mask trends in particular functional groups. We surveyed bee communities along an urban-to-rural gradient in SE Michigan, USA, and document a large change in observed sex ratio (OSR) along this gradient. OSR became more male biased as urbanization increased, mainly driven by a decline in medium and large bodied ground-nesting female bees. Nest site preference and body size mediated the effects of urbanization on OSR. Our results suggest that previously documented negative effects of urbanization on ground-nesting bees may underestimate the full impact of urbanization, and highlight the need for improved understanding of sex-based differences in the provision of pollination services by wild bees.

## Introduction

Wild bees (Apoidea: Hymenoptera) are critically important both to agricultural production and the maintenance of angiosperm biodiversity^1,2^. But populations of many species are in widespread decline^3^ due to multiple interacting factors, including parasites and disease^4^, climate change^3,4^, pesticide use^5^ and habitat loss^5^. Pesticide use and habitat loss in particular, are largely driven by agricultural conversion and intensification^5–7^. Urbanization has also contributed to habitat loss worldwide, evidenced by the increase in the amount of land occupied by urban development in the past 50 years^8,9^ and this trend is expected to accelerate in the coming decades^9^. Less well understood, however, is how urbanization affects bee communities.

Studies examining changes in bee communities along the rural-to-urban gradient have found relatively minor effects on overall abundance and diversity, particularly in comparison to the effects of agricultural intensification^10–12^. However, evaluating only aggregate abundance and diversity masks trends in particular guilds of bees; most notably, studies have consistently found reduced abundance and/or diversity of ground-nesting bees in urban areas^10,12–15^. This has been attributed to the lack of appropriate nesting substrate for ground-nesting bees in urban areas, though reduction in ground-nesting bee nest density or nest site availability has rarely been shown directly (but see ref.^16^). Thus, while the available evidence suggests that urban areas are capable of supporting bee communities^17,18^, it also indicates that these communities are likely to differ systematically from those found outside cities, with, for example, an underrepresentation of ground-nesting bees^10^.

While considering nesting or feeding ecology can reveal differential effects of urbanization on bee communities^10^, using ecological guild or even species as the unit of analysis may obscure other important effects of urbanization on bee communities. In particular, life history differences between female and male bees seem likely to result in distinct trends in observed sex ratio (OSR) with increasing urbanization^19^. There are non-exclusive mechanisms by which urbanization may drive changes in OSR, explored in greater detail below: (1) sex-specific patterns of movement and dispersal, (2) labile sex ratios and (3) temperature.

### Sex-specific movement patterns

For most of their life cycle, non-parasitic female bees are central-place foragers, collecting nectar and pollen in order to provision their brood; as a result, most foraging occurs close to the nest site^20^. Male bees, on the other hand, do not engage in parental care, instead dispersing in search of mates. Moreover, while reproductive females also disperse from the natal nest prior to establishing their own nest, females tend to disperse shorter distances than males^21–23^. In urban landscapes, habitat patches (e.g. community gardens) are fragmented within a built matrix that is likely to be low in suitable nesting sites (at least for ground-nesting bees^10,16^) and floral resources^24^, but see ref.^25^. Sex-based differences in movement patterns, in combination with this high degree of fragmentation, could result in changes to OSRs relative to those seen in more intact landscapes.

### Labile sex ratios

Sex allocation in bees is labile, and dependent in part on (1) food resource availability, with greater food abundance resulting in a higher proportion of female offspring^26,27^, and (2) brood cell parasitism rates, with increased parasitism pressure resulting in reduced provisioning and therefore fewer female offspring^28,29^. Systematic changes in the ability of foragers to provision their brood along the urban-to-rural gradient, resulting from changes in either the abundance or distribution of suitable floral resources or brood parasitism rates, could therefore result in OSR shifts along an urbanization gradient.

### Temperature

Another possible explanation for differences in OSR across an urban-to-rural gradient may be phenological shifts associated with the urban heat island effect. Bee emergence is related to temperature^30^ suggesting that bee phenology will possibly be advanced in more urbanized areas, where temperatures are higher than the surrounding landscape^31^. Moreover, in many solitary bee species, male bees tend to emerge several days earlier than female bees. Since pan-trap sampling occurred on the same day at all sites for each sampling bout, this could lead to shifts in OSR as temperature increases along the urbanization gradient if the sampling date fell during the emergence period of one or more species.

Environmentally-generated spatial variation in OSR in bees has been scarcely investigated. Recent work has documented decreases in female relative abundance in bumble bees (*Bombus* spp.) along a rural-to-urban gradient^19^. Since most bumble bee species are eusocial (or social parasites), sex allocation - and resultant OSR - may be influenced by factors absent in solitary bee populations, including queen-worker genetic conflict^32,33^. Thus, findings from bumble bees cannot necessarily be extrapolated to the wild bee community as a whole, comprised as it is primarily of non-eusocial species. This study represents, to the best of our knowledge, the first investigation of OSR in a complete wild bee community along a land use gradient.

The potential for urbanization to drive changes in OSR is significant for several reasons. First, changes in adult sex ratios can affect population dynamics^34,35^; assuming a constant sex ratio when modeling demographic rates can lead to incorrect conclusions about population trends^34,35^. Second, there is evidence for sex-based differences in bee foraging behavior, including floral preferences^36^, floral constancy (i.e. the tendency to sequentially visit flowers of the same species)^37^, pollen transfer efficiency^38^, and flight distance between foraging bouts^37,38^. Thus, changes to OSR have the potential to impact both bee population dynamics and pollination services.

Here, we document a shift in OSR in bee communities found in community gardens along a rural-to-urban gradient, where the proportion of male bees increases with urbanization. We find that the observed increase in male relative abundance is primarily due to declining absolute abundance of medium- and large-bodied female ground-nesting bees as urbanization increases. We discuss potential mechanisms that may generate the OSR shifts, as well as implications for future research on urban bee communities.

## Results

We caught a total of 3,336 bees (Table S1) consisting of 143 species across 28 genera (Table S2). Of these, 2,481 (74%; 95 species) belonged to species that nest underground (hereafter ‘ground-nesting bees’), while 855 (26%; 48 species) belonged to species that nest above ground in cavities or hollow stems (hereafter ‘cavity-nesting bees’). Ground-nesting bees in the sampled population were comprised of 60.9% eusocial bees, 18.8% solitary bees, and 18.1% bees that either nest communally or exhibit variability in sociality mode (classified as “other” in this paper; see Methods). The remaining 2.2% of ground-nesting bees were cleptoparasites or bees with unknown sociality mode. Cavity-nesting bees were overwhelmingly solitary (96.0%).

The effect of urbanization, as measured by impervious surface cover (hereafter ‘ISC’), on bee OSR was qualitatively similar regardless of the scale at which it was assessed (500 m, 1 km, 1.5 km, or 2 km), but the model assessing urbanization at the 2 km scale had the lowest AIC value (Table 1; ∆AIC = 1.48 for next-best model measuring urbanization at 1.5 km), so this scale was used for subsequent analyses.

**Table 1:** Model comparison for predicting bee observed sex ratio.

Predictor(s)	Residual deviance	Significance - *p*	Effect Size - *β*	AIC
500 m	20.28_24_	(500 m)6.60*e*^−5^	−0.32 ± 0.08	187.38
1 km	17.60_24_	(1 km)1.76*e*^−5^	−0.40 ± 0.09	184.7
1.5 km	14.91_24_	(1.5 km)4.51*e*^−6^	−0.51 ± 0.11	182.01
2 km	13.42_24_	(2 km)2.08*e*^−6^	−0.57 ± 0.12	180.53
2 km + Floral abundance	13.20_23_	(2 km)8.83*e*^−6^ (Abund)0.84	(2 km)−0.57 ± 0.13 (Abund)4.25*e*^−7^ ± 2.13^−6^	182.35
2 km + Floral area	11.97_23_	(2 km)6.71*e*^−6^ (Area)0.26	(2 km)−0.54 ± 0.12 (Area)2.55*e*^−7^ ± 2.26*e*^−6^	181.13
2 km + Richness	13.19_23_	(2 km)1.17*e*^−5^ (Richness)0.82	(2 km)−0.55 ± 0.13 (Richness)2.31*e*^−4^ ± 0.001	182.34

Models were GLMs with poisson distribution and log-link function. The first four rows present the effect of impervious surface cover measured at different scales (i.e. within 500 m of the garden, within 1 km, etc.). Floral metrics are season-long means (abundance and area; averaged over the number of floral surveys) or totals (richness) for an area within 20 m of the study location.

We found residual spatial autocorrelation (SA) in a small number of models; in all other cases calculation of Moran’s I indicated no SA. In cases where SA was detected, inclusion of Moran’s eigenvectors as predictors in the model had only minor effects on estimates and significance levels of other model terms and overall model fit, with no qualitative changes to results (Table S3). Below, where we report model output for a model that had SA, reported values are from the model modified to include Moran’s eigenvectors.

Neither total bee abundance nor the abundance of ground-nesting bees was affected by urbanization (total: *t* = −0.36, d.f. = 24, p = 0.73; ground-nesting: *t* = −1.09, d.f. = 24, p = 0.29), but abundance of cavity-nesting bees increased with urbanization (t = 2.62, d.f. = 24, p = 0.01) (Figure S1). Local floral resource availability, whether measured by total number of blooms, total floral area or floral richness within 20 m of the sampling point, had no effect on bees when season-long average floral resource availability was considered, but positively influenced total bee abundance and richness when survey periods were taken into account (Table 2). While the effects of all 3 floral metrics were qualitatively similar, floral abundance, as measured by the total number of blooms, was the best predictor of bee abundance, while floral richness was the best predictor of bee richness (Table 2). The effects of floral resource availability were similar whether bees were considered in aggregate or with nesting guilds and sexes considered separately, except that floral resource availability was not found to influence the abundance of male cavity-nesting bees (AIC value for model including floral richness was identical to AIC value for model that omitted it; Table 2).

**Table 2:** Relationship between floral resource availability and bee community characteristics.

Relationship between floral resource availability and bee community characteristics													
	Floral abundance				Floral area				Floral richness				Other predictors
	Resid. dev.	z	ß	∆AIC	Resid. dev.	z	ß	∆AIC	Resid. dev.	z	ß	∆AIC	
**All bees**													
Total abundance	**830**.**2**_**96**_	**5**.54***	**0**.**31**	**0**.**0**	846.7_96_	3.09***	0.21	16.5	837.6_96_	4.54***	0.32	7.4	Period
Female abundance	**785**.**3**_**97**_	**5**.89***	**0**.**34**	**0**.**0**	802.6_97_	3.46***	0.23	17.3	794.6_97_	4.62***	0.32	9.3	—
Male abundance	**547**.**8**_**95**_	**3**.16***	**0**.**31**	**0**.**0**	555.1_95_	1.51	0.17	7.3	553.5_95_	1.96	0.22	5.6	%ISC + Period
Richness	597.6_97_	4.97***	0.18	5.2	610.2_97_	3.08**	0.13	17.8	**592**.**4**_**97**_	**5**.57***	**0**.**23**	**0**.**0**	—
**Ground-nesting bees**													
Total abundance	**793**.**0**_**96**_	**5**.10***	**0**.**32**	**0**.**0**	807.0_96_	2.88**	0.21	15.0	799.5_96_	4.11***	0.34	6.5	Period
Female abundance	**745**.**8**_**96**_	**5**.15***	**0**.**33**	**0**.**0**	759.1_96_	3.06**	0.22	14.3	751.7_96_	4.21***	0.32	5.9	%ISC
Male abundance	**474**.**0**_**95**_	**2**.91**	**0**.**35**	**0**.**0**	479.5_95_	1.64	0.21	5.5	477.4_95_	2.21*	0.30	3.4	%ISC + Period
**Cavity-nesting bees**													
Total abundance	**504**.**0**_**83**_	**2**.91**	**0**.**23**	**0**.**0**	510.0_83_	1.32	0.11	6.0	504.6_83_	2.72**	0.23	0.6	%ISC
Female abundance	**461**.**7**_**83**_	**2**.73**	**0**.**24**	**0**.**0**	467.2_83_	1.17	0.11	5.5	463.0_83_	2.40*	0.22	1.3	%ISC
Male abundance	312.5_83_	1.32	0.17	0.3	314.1_83_	0.34	0.05	1.9	**312**.**2**_**83**_	**1**.**44**	**0**.**19**	**0**.**0**	%ISC

Comparison of model output for GLMMs with study site as a random effect and an observation-level random effect included to account for overdispersion. “Other predictors” refers to additional predictors beyond the floral resource metric included in the best model. Boldface indicates output from the best model. %ISC: percent of the landscape covered by impervious surfaces within 2 km of the study site. Significance levels: *p < 0.05, **p < 0.01, ***p < 0.001.

**Figure 1:**
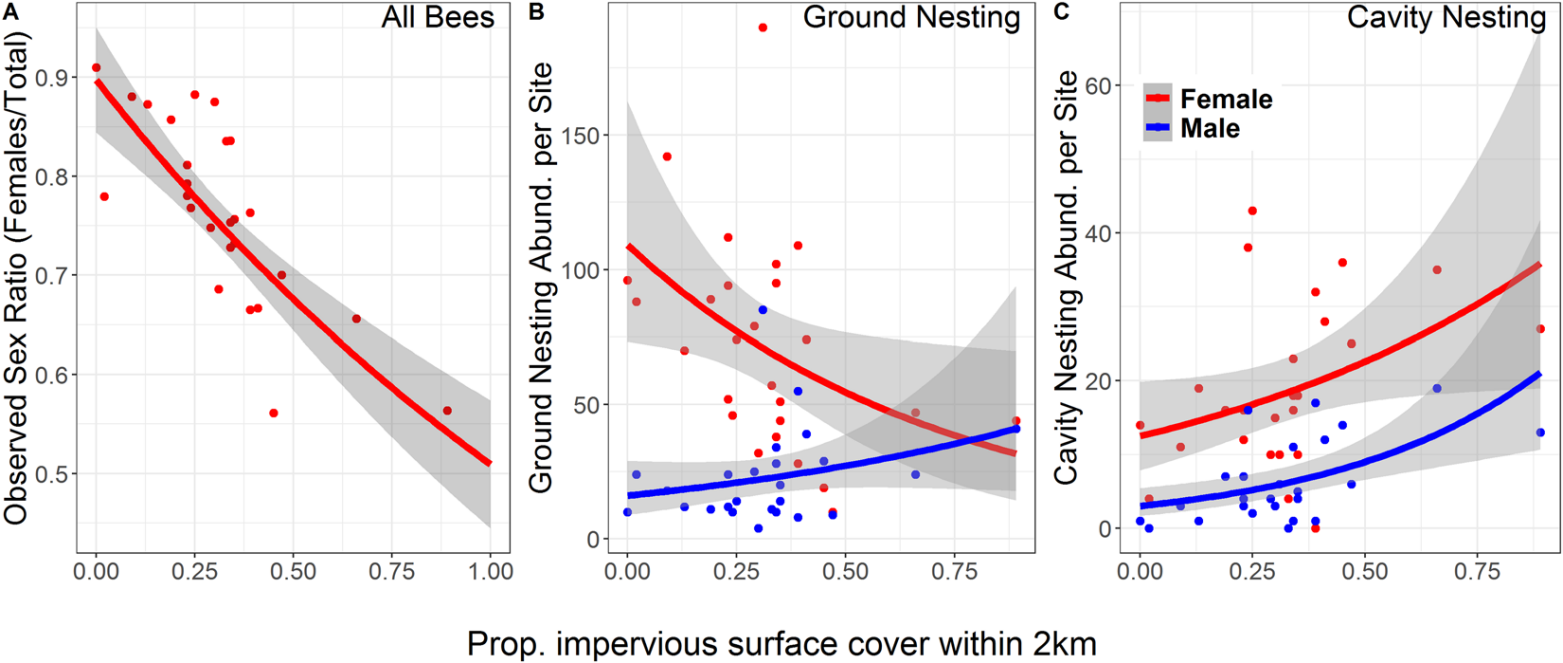
Effects of urbanization on wild bee community. Relationship between the level of urban development (measured as proportional ISC within 2 km of the sampling site) and (**A**) bee observed sex ratio per site (OSR) (z = −4.73, d.f. = 23, p < 0.001); (**B**) ground-nesting bee abundance per site of females (red, t = −2.18, d.f. = 24, p = 0.04) and males (blue, t = 1.41, d.f. = 24,p = 0.17); and (**C)** cavity-nesting bee abundance per site of females (red, t = 1.98, d.f. = 24, p = 0.06) and males (blue, t = 3.36, d.f. = 24, p = 0.003). Fitted line in **A** represents GLM fit of female abundance offset by total abundance; in (**B**,**C**) lines represent GLM fit of female (red) or male (blue) abundance. Shaded regions represent standard error.

No metric of floral resource availability (considered either as season-long averages or separated by period) was a significant predictor of OSR (Tables 1 and 2). Mean minimum temperature was a significant predictor of OSR, though less so than ISC (Table S4) and the significant relationship was drive by mean minimum temperature’s correlation with ISC. When all observations across the survey were combined, the best model for predicting OSR included only urbanization, determined by AIC values, (Table 1). When sampling periods were considered separately, models including floral resource metrics were indistinguishable from the model including only urbanization and sampling period (∆AIC < 1; Table 2). These findings held whether the entire bee community was considered together or nesting guilds were considered independently (Tables S5 and S6). Moreover, there was no relationship between local floral resource metrics and urbanization (Table S7). The observed sex ratio (OSR) of the sample populations changed significantly along the rural-to-urban gradient, with relative abundance of females decreasing with urbanization (*z* = −4.73, d.f. = 23, p < 0.001; Fig. 1A). This overall change in OSR was driven entirely by changes in ground-nesting bees (*z* = −3.66, d.f. = 23, p < 0.001, corrected for SA); in cavity-nesting bees OSR was consistent across the rural-to-urban gradient (*z* = −1.42, d.f. = 23, p = 0.16). The change in OSR in ground-nesting bees is the result of declining female abundance with increasing urbanization (*t* = −2.18, d.f. = 24, p = 0.04); male abundance remained essentially unchanged across the urbanization gradient (*t* = 1.41, d.f. = 24, p = 0.17; Fig. 1B). These results are robust even with the removal of eusocial members of the genus *Bombus* - the most abundant genus of ground-nesting bees - from the dataset (Table S3). By contrast, in cavity-nesting bees, abundance of both sexes increased with urbanization, marginally so in females (*t* = 1.98, d.f. = 24, p = 0.06) and significantly in males (*t* = 3.36, d.f. = 24, p = 0.003; Fig. 1C). These results were robust even when we removed the 2 sites with >50% ISC from the dataset (Table S8), indicating that the relationship between OSR and urbanization occurs with even low-to-moderate degree of urbanization. The decline in female relative abundance in ground-nesting bees was significant and consistent across sociality classes (Table 3) suggesting that shifts in OSR were not related to degree of sociality. This was true despite the smaller number of solitary bees and bees in the ‘other’ category caught relative to eusocial bees. Therefore, the pattern of OSR shift in ground-nesting bees was not driven exclusively by eusocial species.

**Table 3:** Bee observed sex ratio responses to urbanization by sociality class.

Effect of Prop. Impervious Surface Coverage on OSR of Ground Nesting Bees Across Sociality Categories			
Sociality Categories	Residual Deviance	Significance - *p*	Effect Size - *β*
Eusocial	19.07_24_	0.019	−0.385 ± 0.164
Other	18.66_22_	0.048	−1.001 ± 0.507
Solitary	16.77_24_	0.003	−0.994 ± 0.334

Model for Eusocial bees was corrected for SA (Table S3).

The relationship between urbanization and OSR in ground nesters is mediated by body size: there was no effect of urbanization on OSR in small ground-nesting bees (*z* = −0.686, d.f. = 24, p = 0.49); while both medium and large ground-nesting bees experienced decreases in female relative abundance with increasing urbanization. The effect of urbanization on OSR was stronger for large than medium bees [large: *z* = −3.79, d.f. = 23, p < 0.001 (corrected for SA); medium: *z* = −3.07, d.f. = 24, p = 0.002; Figure S2].

**Figure 2:**
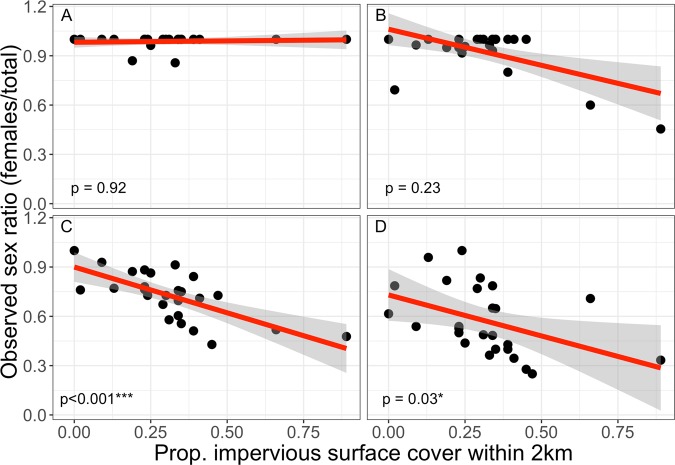
Relationship between wild bee observed sex ratio (OSR) in ground nesting bees and urbanization across the flying season. Each period includes one bout of netting and two flanking bouts of pan trapping. (**A**) Period 1: 19 May – 5 June, (**B**) Period 2: 19 June – 2 July, (**C**) Period 3: 17 July – 13 August, (**D**) Period 4: 26 August – 26 September. The p-values refer to the effect of ISC on OSR. Shaded region represents standard error.

No change in OSR was seen across the season for cavity-nesting bees (z = −0.872, d.f. = 86, p = 0.383). In ground-nesting bees, OSR changed significantly across the season, with the proportion of female bees caught declining at each successive sampling period (z = –8.345, d.f. = 99, p < 0.001). Moreover, correlation between ground nesting OSR and urbanization was seen only in the second half of the survey season; periods one (19 May–5 Jun) and two (19 Jun–2 Jul) show no significant change in OSR across the rural-to-urban gradient (Fig. 2).

## Discussion

Here we document a shift in observed sex ratio (OSR) of ground-nesting bees along an urbanization gradient, with the relative abundance of female bees declining as urbanization increases while the abundance of male ground-nesting bees remained unaffected by urbanization. Because provisioning female bees tend to focus foraging efforts in the vicinity of their nest^20^, female abundance is likely to be correlated with local nest density. Therefore, these data suggest that urbanization reduces nest density of ground-nesting bees, consistent with findings from other researchers^10,12,13^, and that this generates the observed OSR shift in ground-nesting bees. A key question, then, is why we do not see a parallel decline in male ground-nesting bees with increasing urbanization. One possible explanation is that, because male bees are not tied to a nest, they may be disproportionately abundant in floral resource-rich areas even if nest density in these areas is low. Male bees also tend to disperse longer distances than dispersing reproductive females^21–23^. Sampling sites for this study were located in community gardens, which tend to have higher density and diversity of floral resources than the surrounding landscape^39^ and the disparity between within-garden and outside-garden floral resource availability increases with urbanization in the study region^39^. This pattern could lead to a disproportionate concentration of male bees at more urban sampling sites. Our finding that urbanization-associated changes in ground-nesting bee OSR occur only among medium- and large-bodied bees further supports this explanation. Movement distance in bees is strongly correlated with body size^40^; thus, males of larger species are more likely to disperse sufficiently far from their natal nest to reach resource patches in urban landscapes. The sample of smaller-bodied bees, on the other hand, more closely reflects the makeup of the locally-originating population.

An alternative explanation for the observed OSR shift could be urbanization-induced changes in sex allocation by bees. Specifically, it is known that sex allocation in bees can be influenced by floral resource availability^26,27^ and abundance of brood parasites^28,29^, as both factors influence provisioning of brood cells^27,28^. In most bee species, the production of reproductive females requires greater resource investment than the production of males; consequently, reduced maternal provisioning may result in a shift towards production of males^27,28^. In eusocial species, production of workers (which are female but do not reproduce), is also correlated with resource availability^41^. Thus, the observed decrease in female relative abundance with urbanization could be the result of reduction in the production of females due to reduced floral resource availability in urban landscapes. Our finding that OSR is influenced by urbanization for only medium and large bees is potentially consistent with either sex allocation or dispersal-based explanations for OSR shifts: larger bees are likely to both have larger foraging ranges (and thus be more affected by floral resource availability in the wider landscape, potentially leading to increased production of males in resource-scarce landscapes) and disperse greater distances (allowing for disproportionate concentration of dispersing males in urban habitat patches). However, effects of floral resource scarcity should not depend on bee nesting strategy; the fact that the OSR shift was found only in ground-nesting bees argues against a resource-mediated shift in sex allocation. Moreover, floral surveys revealed no relationship between urbanization and local (20 m) floral resource availability. Study sites were located within community gardens, which in our study area tend to have higher floral abundance and richness than the surrounding landscape^39^, so the lack of correlation between local floral resource availability and urbanization does not preclude the possibility that landscape-scale floral resource availability was negatively correlated with urbanization; we did not assess landscape-scale floral resource availability in this study. However, the high diversity and abundance of floral resources found within garden study sites likely attenuates the effect of landscape-level floral resource availability^42^. In contrast, parasitism rates may depend on nesting strategy, with ground-nesting bees likely experiencing higher parasite pressure^43,44^. This is consistent with our finding that OSR shifts occurred only in ground-nesting bees, but does not offer any immediate explanations as to why OSR shifts were not apparent in small ground nesting bees. While it is possible that parasite pressure along the urbanization gradient contributes to OSR shifts, we did not directly assess parasite abundance in this study nor are we aware of any study that assesses brood cell parasitism rates along an urban gradient. Further research on the environmental drivers of brood parasitism in bees is needed before we can reach conclusions about the role of parasitism in the observed OSR shift.

We also considered temperature as one of the potential mechanisms driving shifts in OSR. Urbanization and average daily minimum temperature are significantly correlated but the data available does not support temperature as a mechanism for shifts in OSR. While temperature did significantly correlate with the changes seen in overall OSR, this is due to the aforementioned correlation between temperature and urbanization. Temperature also had a distinctly less favorable AIC score when compared to urbanization (Table S4). Additionally, if higher temperatures were causing earlier emergence of males in urban sites, then it would be reasonable to expect that OSR would be more male biased earlier in the season, which we did not find (Fig. 2). The lack of pattern in the OSR of cavity-nesting bees further weakens the case for temperature as one would expect similar changes as those seen in the OSR of ground nesting bees if ambient temperature was driving the OSR shift.

Our finding that bee abundance and richness are positively related to local floral resource availability is consistent with other studies of the determinants of bee community composition in urbanized landscapes^14,45,46^. This is true despite the fact that the scale at which floral resources were sampled is far smaller than the foraging range of most bees. These findings highlight the importance of floral resource-rich habitat patches for conserving wild bee populations, particularly in highly fragmented landscapes.

Finally, we found that the abundance of both male and female cavity nesting bees increased with urbanization. This is an anticipated result given that a number of studies have found similar results for cavity-nesting bees^10,12,13,45–48^. This may be due to the presence of anthropogenic cavities in urbanized habitats providing nesting resources for cavity-nesting bees^12,49^ in contrast to ground-nesting bees. Cavity-nesting bees may also benefit from a reduction in competition for floral resources due to the reduced abundance of female ground-nesting bees.

Our findings highlight the importance of considering sex-specific differences in bee behavior when analyzing the effects of environmental change on bee populations. Even though our results pertain to just one year of sampling and interannual variation may affect the degree of change in OSR, they suggest that research may be underestimating the negative impacts of urbanization on ground-nesting bees. While multiple studies have found reductions in ground-nesting bee populations in urban areas^10,12,13^, the magnitude of these reductions may be greater than what total abundance measures indicate if, as we suggest in this study, urban ground-nesting bee populations are subsidized by males dispersing from less urban areas. Further research in other urban areas is needed to determine the generality of the trend we document here, and to conclusively distinguish among the potential mechanisms driving urbanization-related OSR shifts in ground-nesting bees. Finally, these results stress the need for improved understanding of how sex-specific behavior in bees, including patterns of floral preference and pollen transfer efficiency, affect pollination services. At this point, while we know enough to suspect that these differences may be substantial^36–38^, further research is needed to predict the effects of a local shift in bee community sex ratio on plant communities.

## Methods

### Study location

Sampling occurred at 26 sites distributed along a rural-to-urban gradient in southeastern Michigan, USA (Figure S3). Sites spanned a distance of 110 km, with the surrounding land use ranging from dense urban core to suburban to rural-agricultural. 21 of 26 sites were community gardens, 3 sites were nature reserves, and the remaining 2 sites were rural farms. The gardens or farms sampled in each city were either part of an independent managing organization, single home owner properties or property of the University of Michigan (see Table S9). All gardens and farms included in the study observe organic growing practices prohibiting the use of synthetic pesticides and fertilizers.

### Pollinator sampling

Bee fauna were sampled from 19 May to 25 September 2014, using pan traps and active netting. This combination of sampling techniques is widely used in studies of bees, and has been shown to thoroughly sample bee communities^50^.

For pan trapping, 2 oz (59 mL) plastic cups (Dart Container Corporation, Mason, MI USA) coated with UV-reflective paint in one of three colors (white, yellow, and blue), were filled with water and a small amount of soap as a surfactant. Pan traps were placed at all sites once every two weeks for a total of 9 trapping dates. Sampling occurred only on days that were sunny or partly sunny, with wind speeds below 4 m/s. On sampling days, pan traps were placed in all 26 sites before 1000 h and left for 24 h. Pan traps were then removed and all trapped arthropods were placed in 70% ethanol for later processing. Within each site, two pan traps of each color (6 total) were arranged in an 4 m × 2 m rectangle with a pan trap placed at each vertex and middle of the longer sides of the rectangle. This arrangement of pan traps is more compact than that used by some studies, and was devised to accommodate the small areas we were often granted access to sample in (e.g. one plot within a community garden) and to keep our sampling standardized across the study area. To maintain visibility to bees and accommodate changing vegetation heights over the course of the sampling period, pan traps were affixed to adjustable-height PVC pipes, and positioned to be 5–10 cm above surrounding herbaceous vegetation height.

Netting at each site occurred 4 times over the sampling season, once a month from May-September. To account for variation in diurnal activity patterns across species, each sampling event comprised two 30-minute sessions, one between 0900–1200 h and another between 1300–1600h, with the same requirements on meteorological conditions as for pan trapping (see above). Netted bees were transferred to vials containing 70% ethanol for later processing.

All bees were identified to species and assigned to sex. Identification was accomplished using the Discoverlife key^51^, with additional identifications made by Dr. Jason Gibbs (University of Manitoba, Winnipeg, Canada) and Jamie Pawelek (Wild Bee Garden Design, formerly University of California Berkeley, USA). Specimens are housed at the University of Michigan Museum of Zoology (accession numbers UMMZI-99924 through UMMZI-103259).

### Pollinator natural history and body size data

Once all specimens were identified to species, natural history profiles were compiled for each species using four characteristics: preferred nesting substrate, sociality, native status, and body size (Table S2). Most natural history data were generously provided by Dr. Jason Gibbs, supplemented as necessary with literature searches. Bees with known modes of sociality were placed in one of four categories: eusocial, solitary, cleptoparasitic, and other. The ‘other’ category includes species that (1) exhibit both solitary and eusocial strategies either within or across populations or (2) nest communally (‘solitary social’ and ‘communal’ designations in Table S2). Analyses across sociality showed no pattern for cleptoparasites or bees with unknown sociality. However, our sample number of bees in these categories was very low, which affected statistical power. As a measure of body size, we used female intertegular (IT) distance, which is strongly correlated with flight ability and is therefore a proxy measurement of bee dispersal ability and foraging distance^40^. When IT distance could not be found in the literature^52^, we measured IT distance of 5 females of that species from our collection and took the mean as the species-specific IT distance. In cases where the species was represented by fewer than 5 individuals, we took measurements from all available samples; in general variance in IT distance across conspecific individuals was small (Table S2). We only measured IT distances of workers for eusocial species. Bees were then classified as small (≤1.5 mm), medium (>1.5–3.0 mm), or large (>3.0 mm) on the basis of IT span.

### Landscape-level impervious surface measurements

We used National Land Cover Database (NLCD) data from 2011^53^ to calculate the amount of urban development surrounding each study site as described in ref.^19^. Briefly, we used proportion of ISC as our measure of urbanization, and measured ISC at radii of 500 m, 1 km, 1.5 km, and 2 km around the study site. The NCLD classifies land cover in 30 m cells; summing cells within the relevant radius categorized as high- or medium-intensity developed gives the total area of impervious surfaces within each buffer (Table S10).

We used GLMs with Poisson distribution and log-link function to determine the radius at which ISC had the most explanatory power over bee observed sex ratio (OSR), and which, therefore, to include in subsequent analyses. For each radius, we fit a model with overall OSR as the response variable, and proportional ISC at the radius of interest as the sole predictor. We used AIC values to select the best radius.

### Local floral resource and temperature measurements

Floral resource availability within 20 m of the center of pan trap placement was measured at each pan trap sampling date. We identified all plants in flower within this circle to species or morphospecies, and recorded the number of open blooms on each species using a modified logarithmic scale (1–10 blooms, 11–50, 51–100, 101–200, 201–500, 500–1000, >1000). Species-specific flower dimensions were recorded in the field, and per-flower area calculated, as in ref.^4^. Per-species floral area at a given survey was then calculated by multiplying floral abundance (mean value of the abundance bin) by flower size. Summing counts and area for all species gives the overall floral abundance and area, respectively, at each site per sampling date. Mean floral count and area, and total floral richness were calculated for each survey period (see *Analysis*) and for the entire season (Table S11), and we used these metrics to assess the effect of floral resources on the bee community.

Temperature at each site was measured by data loggers (HOBO, Onset Computing Corporation, Bourne, MA USA) placed in an unshaded area within the floral survey circle. Data loggers remained throughout the sampling season and recorded daily average, minimum and maximum temperatures every 24 hours. Because data loggers at several sites were compromised, temperature data were available for 22 of 26 sites (Table S9). While mean minimum temperature had a significant effect on OSR (*z* = *−*2.92, d.f. = 20, p = 0.003), it was also significantly correlated with ISC (e.g. p < 0.001 at 2 km radius). The direction and magnitude of the effects of temperature and ISC were similar, and the model including ISC had a lower AIC value (ΔAIC = 5.43). Thus, we omitted any measure of temperature from the analyses described below; including mean minimum temperature in our models had little impact on model outcomes (Table S4).

### Analysis

All analyses were carried out in R v.3.4.1^54^. Because we were interested in the response of wild bees to urbanization, we excluded records of the managed European honey bee (*Apis mellifera*) from our analysis; *A*. *mellifera* represented 4.9% of collected bees (164 individuals).

The OSR was found by adding together the number of female bees of all species collected at a site, then dividing this by the total number of bees collected at that site. For analysis of the relationship between floral resources and OSR only, OSR at each site was calculated for each sampling period, rather than for the entire season. To model the relationship between OSR and environmental variables, we used GLMs with Poisson distribution and log-link function. To avoid the difficulties of interpretation when modeling ratios, we used number of female bees as our response variable, with log(total bee abundance) included as an offset. Predictor variables in the maximal model included: ISC within 2 km, season-long average floral area within 20 m and season-long total floral richness within 20 m. To test the relationship between OSR and each predictor, we conducted stepwise reduction of the model, beginning with the predictor showing the least explanatory power. The best model was then selected using AIC comparison. Models were checked for overdispersion, and in all cases the dispersion parameter value was <1.4.

Because floral resource availability varied idiosyncratically across sites over the study duration, we evaluated the relationship between floral resource availability and bee community measures for each of 4 periods (in addition to the season-long averages outlined above). Each period consisted of a net-sampling event and the two bracketing pan-trapping events, and had a duration of 3–4 weeks. To model this relationship, we used GLMMs with site as a random effect, and an additional observation-level random effect to account for overdispersion. The maximal model for each bee community measure (i.e. abundance or OSR) in this case included a floral resource metric (abundance, area, or richness), survey period, and ISC. Because of strong collinearity among the 3 floral metrics, a separate model was fit for each metric. Model selection proceeded as outlined above. Since OSR was not affected by floral resource availability (see Results), further investigation of the association between OSR and environmental or bee community attributes was conducted using season-long averages and GLMs, as described in the previous paragraph, rather than data separated by period.

Particularly when we increased the radius at which impervious surface was considered, there was some overlap in study sites. To account for this, we assessed spatial autocorrelation (SA) in all models by calculating Moran’s I for the residuals of all models. In cases where residuals showed significant SA, we used the Moran eigenvector approach^55^, implemented with the ‘ME()’ function in R package ‘spdep’^56^ to adjust the model to account for SA. This approach accounts for SA through the addition of one or more orthogonal eigenvectors describing the spatial structure in the data as model predictor term(s).

Only 2 of 26 sites in the study had >50% ISC at the 2 km scale. To test whether these 2 sites had undue influence on our results, we conducted an identical analysis to that described above after removing observations from those sites from the dataset (Table S8).

To assess whether patterns in OSR differed across the season, we fit a model similar to those outlined for overall OSR, but with the addition of period and a period × ISC interaction as predictors. Because this model indicated both a significant effect of period and a significant period × ISC interaction (see Results), we then fit a separate model for each period, with ISC at the 2 km range the sole predictor.

To determine whether OSR response to urbanization was significantly affected by species attributes (i.e. preferred nest substrate, body size, and sociality), we constructed GLMs that included urbanization, species attribute of interest, and an urbanization × species attribute interaction term as predictors; a significant interaction term indicated significant differences among species in OSR response to urbanization, mediated by the attribute of interest. When a significant interaction was found, we divided the data by attribute of interest (e.g. ground-nesting vs. cavity-nesting; small, medium, or large bees), then ran a separate model for each guild to assess how the relationship between OSR and urbanization varied across guilds.

A parallel analysis was conducted for bee abundance, with model form, predictors, and model selection process as above, with two exceptions. First, because we were looking at abundance, rather than OSR, these models omitted the offset term. Second, abundance data were in all cases significantly overdispersed; to account for this overdispersion, a quasi-Poisson distribution was used in place of the Poisson distribution. Because AIC values cannot be calculated from quasi- distributions, we instead used the related quasi-AIC metric for model comparison.

Bumble bees (*Bombus* spp.) made up a large portion of our sample set; most bumble bee species are eusocial, and as such may experience controls on OSR that differ substantially from other bees (see Introduction). In order to verify that changes in the OSR were not driven solely by bumble bees, additional analyses of OSR changes in ground nesting bees and urbanization were done with bumble bees removed. Analyses show no qualitative differences with bumble bees removed: OSR still becomes less female-biased as urbanization increases (Table S3).

We assessed the relationship between metrics of floral resource availability and urbanization using GLMs with log-link function. As with bee abundance data, floral richness and area metrics were significantly overdispersed, so quasi-Poisson distributions were used to account for overdispersion.

## Supplementary information

Supplementary Information

Table S1

Table S2

Table S10

Table S11

## Data Availability

All data from the analysis presented here is including as Supplementary Data files with this publication.
