# Supplementary material for: Changes in adult sex ratio in wild bee communities are linked to urbanization
Source: Sci Rep. 2019 Mar 6;9:3767. doi: 10.1038/s41598-019-39601-8 (PMC6403428; doi:10.1038/s41598-019-39601-8)
Supplement: Supplementary file 1 — Supplementary Information [file 41598_2019_39601_MOESM1_ESM.pdf]

Supplementary Information Title Page

Changes in adult sex ratio in wild bee communities are linked to urbanization

First Authors

Gordon Fitch[1], *Paul Glaum[1] (corresponding author), Maria-Carolina Simao[2], Chatura Vaidya[1]

Contributing Authors:

Jill Matthijs[3], Benjamin Iuliano[4], Ivette Perfecto[3]

* prglaum@umich.edu

1 – Department of Ecology and Evolutionary Biology University of Michigan 1105 North University Ann Arbor, MI 48109 USA, 734-764-1446

2 – New Jersey Agricultural Experiment Station Rutgers University 88 Lipman Dr, New Brunswick, NJ 08901

3 – School of Environment and Sustainability University of Michigan 830 North University 440 Church St, Ann Arbor, MI 48109

4 – University of Wisconsin, Wisconsin Energy Institute 1552 University Ave Madison, WI 53726

Supplementary Information

Three Supplementary Figures and eleven Supplementary Tables of data and additional analysis.

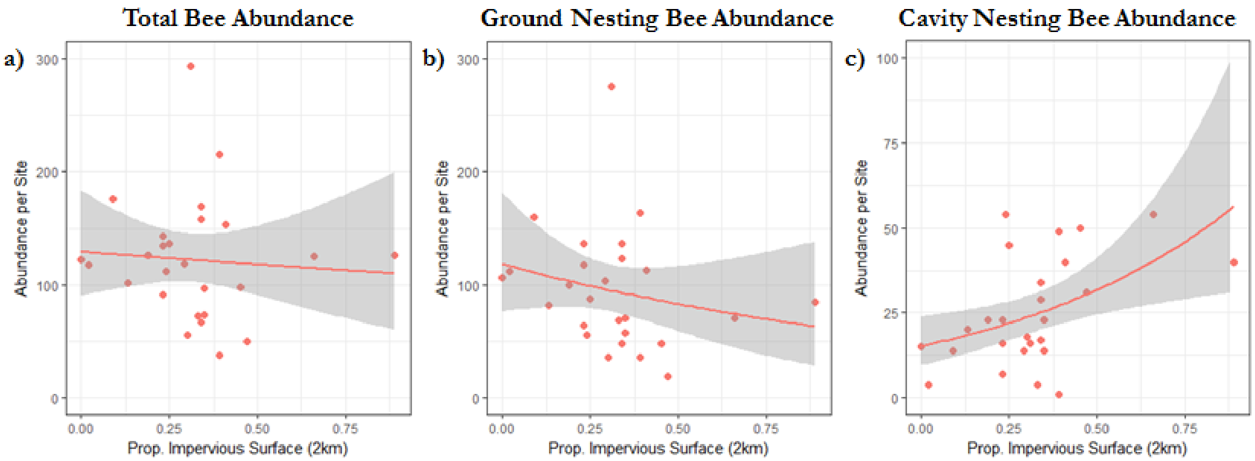

Figure S1: **Relationship between urbanity and bee abundance.** Urbanity was measured through proportional impervious surface coverage within a 2km radius of study site. Regressions were done using GLM with quasi-Poisson distribution. a) Total bee abundance: t= -0.357, df=24, β= -0.1796, p=0.724. b) Ground nesting bee abundance: t= -1.087, df=24, β= -0.7033, p= 0.288. c) Cavity nesting bee abundance: t= 2.712, df=24, β= 1.470, p=0.012.

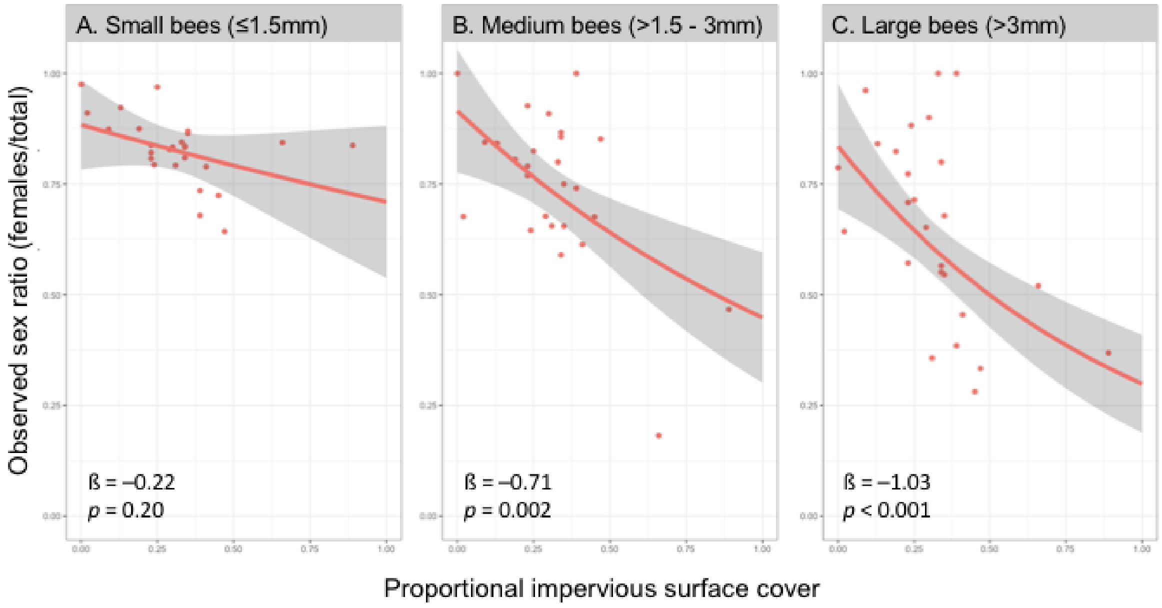

Figure S2: **Relationship between urbanity and bee observed sex ratio as mediated by body size.** Body size was measurements are taken from the intertegular distance, which is a

proxy for flight distance. Urbanity measured as proportional impervious surface cover within 2km of the study site. Line represents best fit for GLM model of number of female bees offset by total number of bees, using Poisson distribution and log-link; shaded area represents standard error. *z*-scores for each size class are as follow: a) small bees, $z = -1.28$; b) medium bees, $z = -3.09$; c) large bees, $z = -4.06$; in all cases d.f. = 24.

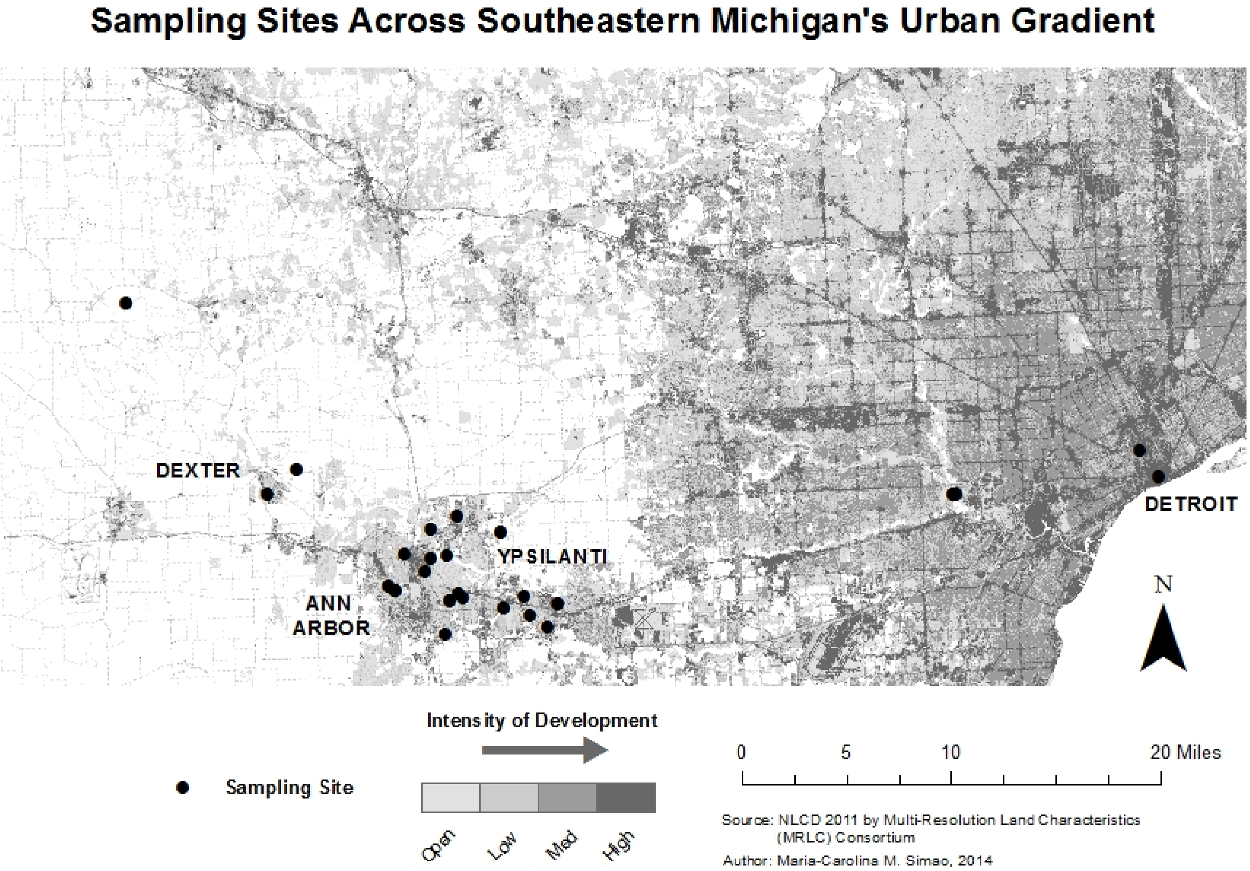

Figure S3. Black dots represent sampling sites used in our study from the rural to urban gradient in S.E. Michigan. Degree of urbanization is depicted by light grey (low ISC) to dark grey (high ISC) colours.

Table S1: **List of individual bee specimens.** (see attached file)

Table S2: **List of bee species collected with natural history information.** (see attached file)

Table S3: **Model comparison for spatially autocorrelated models with (spatial) and without (non-spatial) Moran's eigenvectors included to account for spatial autocorrelation.** *p < 0.05, **p < 0.01, ***p<0.001.

Model	Non-spatial			Spatial		
	β	Res. dev.	AIC	β	Res. dev.	AIC
**Ground nester OSR ~ urbanization**	-0.644 ± 0.140***	16.487_{24}	175.4	-0.568 ± 0.155***	15.206_{23}	176.1
**Eusocial ground nester OSR ~ urbanization**	-0.385 ± 0.164*	19.069_{24}	167.3	-0.365 ± 0.164*	17.577_{23}	167.8
**Large ground nester OSR ~ urbanization**	-1.151 ± 0.281***	26.779_{24}	141.0	-1.242 ± 0.327***	26.473_{23}	142.7
**Non-*Bombus* ground nester OSR ~ urbanization**	-0.588 ± 0.162***	7.327_{24}	160.8	-0.575 ± 0.162***	5.651_{22}	163.2

Table S4: **Regressions against observed sex ratio at sites with temperature measurements. Regressions only include sites where temperature data loggers were able to be retrieved (see Table S9).**

Effect on female ratio	Residual deviance	Significance - p	Effect Size - β	AIC
500m	12.99_{20}	(500m) 0.001	-0.30 ± 0.09	155.01
1km	10.79_{20}	(1km) $4.4e^{-4}$	-0.40 ± 0.11	152.81
1.5km	9.67_{20}	(1.5km) $2.2e^{-4}$	-0.50 ± 0.14	151.69
2km	10.52_{20}	(2km) $3.2e^{-4}$	-0.55 ± 0.15	152.54
Temperature	15.01_{20}	(Temp)0.004	-0.02 ± 0.01	157.12
2km + Temperature	10.47_{19}	(2km)0.03<br>(Temp)0.82	(2km)-0.51 ± 0.24<br>(Temp)$-0.002 \pm 8e^{-3}$	154.49
2km + Floral abundance	10.28_{19}	(2km)$4.07e^{-4}$<br>(Abund)0.83	(2km)-0.56 ± 0.16<br>(Abund)$-4.94e^{-7} \pm 2.3e^{-6}$	154.36
2km + Floral area	9.56_{19}	(2km)$5.48e^{-4}$<br>(Area)0.38	(2km)-0.53 ± 0.15<br>(Area)$2.28e^{-7} \pm 2.6e^{-7}$	153.64
2km + Floral richness	9.39_{19}	(2km)0.007<br>(Richness)0.33	(2km)-0.47 ± 0.17<br>(Richness)-0.001 ± 0.001	153.46

Table S5: **Model comparison for predicting bee observed sex ratio in ground nesting bees**. The first four rows present the effect of impervious surface cover measured within circles of increasing radii (i.e. within 500m of the garden, within 1km, etc.).

Effect on female ratio	Residual deviance	Significance - p	Effect Size - β	AIC
500m	24.92_{24}	(500m)$2.7e^{-4}$	-0.35 ± 0.10	183.81
1km	22.26_{24}	(1km)$7.01e^{-5}$	-0.44 ± 0.11	181.15
1.5km	18.90_{24}	(1.5km)$1.26e^{-5}$	-0.55 ± 0.13	177.78
2km	16.48_{24}	(2km)$3.71e^{-6}$	-0.65 ± 0.14	$\underline{175.36}$
2km + Floral abundance	16.29_{23}	(2km)$3.02e^{-5}$<br>(Abund)0.66	(2km)-0.62 ± 0.15<br>(Abund)$1.1e^{-6} \pm 2.5e^{-6}$	177.21
2km + Floral area	15.85_{23}	(2km)$1.38e^{-5}$<br>(Area)0.42	(2km)-0.62 ± 0.14<br>(Area)$2.06e^{-7} \pm 2.6e^{-7}$	176.78
2km + Floral richness	16.46_{23}	(2km)$1.38e^{-5}$<br>(Richness)0.86	(2km)-0.65 ± 0.15<br>(Richness)$2.19e^{-4} \pm 0.001$	177.38

Table S6: **Model comparison for predicting bee observed sex ratio in cavity nesting bees**. The first four rows present the effect of impervious surface cover measured within circles of increasing radii (i.e. within 500m of the garden, within 1km, etc.).

Effect on female ratio	Residual deviance	Significance - p	Effect Size - β	AIC
500m	8.47_{24}	(500m)0.11	-0.25 ± 0.16	128.45
1km	8.31_{24}	(1km)0.10	-0.30 ± 0.18	128.28
1.5km	8.60_{24}	(1.5km)0.12	-0.34 ± 0.22	128.57
2km	9.02_{24}	(2km)0.16	-0.35 ± 0.24	129.00
2km + Floral abundance	8.77_{23}	(2km)0.14<br>(Abund)0.61	(2km)-0.37 ± 0.25<br>(Abund)$-2.11e^{-6} \pm 4.2e^{-6}$	130.74
2km + Floral area	8.58_{23}	(2km)0.16<br>(Area)0.50	(2km)-0.34 ± 0.24<br>(Area)$3.31e^{-7} \pm 4.95e^{-7}$	130.55
2km + Floral richness	8.55_{23}	(2km)0.22<br>(Richness)0.49	(2km)-0.31 ± 0.25<br>(Richness)-0.001 ± 0.002	130.52

Table S7: **Relationship between urbanity and floral resource availability.**

Relationship between Impervious Surface (2km) and Floral Resources			
**Floral resource metric**	**Residual dev.**	***t***	***p***
Season-long mean abundance	23.07_{24}	–1.42	0.17
Season-long mean area	24.79_{24}	–0.46	0.65
Season-long total richness	23.28_{24}	1.33	0.20

Table S8**: Results of GLMs assessing the relationship between bee observed sex ratio (OSR) and impervious surface cover within 2km of survey sites, including only sites with <50% impervious surface cover.** *p < 0.05, **p < 0.01, ***p<0.001.

	**<50% impervious surface sites**			**All sites**		
**Response variable**	β	**z**	**Residual deviance**	β	**z**	**Residual deviance**
**All bee OSR**	-0.67 ± 0.17 ***	-3.88	12.46_{22}	-0.56 ± 0.12 ***	-4.73	13.29_{24}
**Ground nesters OSR**	-0.71 ± 0.19 ***	-3.70	15.87_{22}	-0.64 ± 0.14 ***	-4.60	16.49_{24}
**Cavity nesters OSR**	-0.52 ± 0.43	-1.19	8.63_{22}	-0.34 ± 0.24	-1.42	9.02_{24}

Table S9: **Site characteristics.**

Site Name	Initials	City Group	Managing Organization	Temperature Data Logger
Arboretum	A	Ann Arbor	U of M	Yes
Boehnke Household	BH	Ann Arbor	Independent	Yes
Buhr Park	B	Ann Arbor	Project Grow	No
Cultivating Community	CC	Ann Arbor	U of M	Yes
Clague Elementary	CE	Ann Arbor	Project Grow	Yes
Campus Farm	CF	Ann Arbor	U of M	Yes
County Farm Park	CFP	Ann Arbor	Project Grow	Yes
Ellsworth	E	Ann Arbor	Project Grow	Yes
Greenview	GV	Ann Arbor	Project Grow	Yes
Leslie Science Center	LSC	Ann Arbor	Project Grow	Yes
Platt	P	Ann Arbor	Project Grow	Yes
Scio Church	SC	Ann Arbor	Independent	Yes
SPH Garden	SPH	Ann Arbor	U of M	Yes
West Park	WP	Ann Arbor	Project Grow	Yes
Organic Garden	OG	Dearborn	U of M	Yes
Old Field	OF	Dearborn	U of M	No
Lafayette Greens	LG	Detroit	Independent	Yes
N. Cass Community Garden	NC	Detroit	Independent	No
Dexter Community Garden	DCG	Dexter	Independent	Yes
E.S. George Reserve	ESG	Dexter	U of M	Yes
M'Lis Farm	MF	Dexter	Independent	Yes
Catholic Social Services	CSS	Ypsilanti	Growing Hope	Yes
EMU - The Giving Garden	EMU	Ypsilanti	Growing Hope	Yes
Frog Island Community Garden	FI	Ypsilanti	Growing Hope	Yes
Normal Park Community Garden	NP	Ypsilanti	Growing Hope	Yes
Perry / Parkridge Community Garden	PCG	Ypsilanti	Growing Hope	No

Table S10: **List of proportional impervious surface coverage around each site.** (see attached file)

Table S11: **List of floral data measured at each site.** (see attached file)
